# A Multiscale Approach to the Smart Deployment of Micro-Sensors over Lightweight Structures

**DOI:** 10.3390/s17071632

**Published:** 2017-07-15

**Authors:** Giovanni Capellari, Francesco Caimmi, Matteo Bruggi, Stefano Mariani

**Affiliations:** 1 Dipartimento di Ingegneria Civile e Ambientale, Politecnico di Milano, Piazza L. da Vinci 32, 20133 Milano, Italy; giovanni.capellari@polimi.it (G.C.); matteo.bruggi@polimi.it (M.B.); 2 Dipartimento di Chimica, Materiali e Ingegneria Chimica “Giulio Natta”, Politecnico di Milano, Piazza L. daVinci 32, 20133 Milano, Italy; francesco.caimmi@polimi.it

**Keywords:** structural health monitoring, damage detection, sensor network, topology optimization, multiscale analysis, microelectromechanical systems

## Abstract

A topology optimization approach has been recently proposed to maximize the sensitivity to damage of measurements, collected through a network of sensors to be deployed over thin plates for structural health monitoring purposes. Within such a frame, damage is meant as a change in the structural health characterized by a reduction of relevant stiffness and load-carrying properties. The sensitivity to a damage of unknown amplitude and location is computed by comparing the response to the external actions of the healthy structure and of a set of auxiliary damaged structures, each one featuring reduced mechanical properties in a small region only. The topology optimization scheme has been devised to properly account for the information coming from all of the sensors to be placed on the structure and for damage depending on its location. In this work, we extend the approach within a multiscale frame to account for three different length scales: a macroscopic one, linked to the dimensions of the whole structure to be monitored; a mesoscopic one, linked to the characteristic size of the damaged region; a microscopic one, linked to the size of inertial microelectromechanical systems (MEMS) to be used within a marginally-invasive health monitoring system. Results are provided for a square plate and for a section of fuselage with stiffeners, to show how the micro-sensors have to be deployed to maximize the capability to detect a damage, to assess the sensitivity of the results to the measurement noise and to also discuss the speedup in designing the network topology against a standard single-scale approach.

## 1. Introduction

Civil and aeronautical structures are continuously exposed to external actions, represented not only by mechanical loads, that can be predicted at the design stage only in statistical terms. Progressive aging, whose time evolution cannot be ascertained deterministically, introduces a further source of uncertainty into the lifecycle management of the structures. It thus seems compulsory to think about structural health monitoring (SHM) systems [1] pervasively diffused or embedded into the hosting structures. In this way, even without resorting to functional materials able to react in real time to simultaneously-varying external conditions and structural properties [2,3], some degree of smartness can be featured by the resulting structures if they become able to detect variations in their own health. This would be of paramount importance especially if a capability to act against the aforementioned detrimental effects of damaging processes helped save lives.

In this paper, we focus on some aspects of the self-sensing capability of smart structures; issues related to self-actuation systems, preventing the initiation and/or growth of defects possibly causing local or global structural failures are not considered. The embedment of the sensing capability into the hosting structure requires the deployment of a sensor network all over the regions exposed to damage processes. Within the proposed frame, a damage is defined as a degradation of the load-carrying capacity of the structure and can be represented by a reduction of the local material strength or stiffness due to environmental conditions backed by physical loading. Damage can be also linked to a reduction of geometric features with a role in defining the overall strength and stiffness characteristics of a structural component. With reference to lightweight composite structures (or laminates), a reduction of the mechanical properties is usually due to intra-laminar damage processes, whereas a reduction of the geometrical properties can be linked to an inter-laminar damage inducing the decohesion of adjacent laminae [4,5,6,7]. In all of the cases, the physical processes are ultimately governed by micromechanical features of the considered multi-phase materials. As extensively discussed in the literature [8,9,10], such events may be difficult to detect in real time as they usually get intercepted and grow inside the laminate and are therefore hidden by the structural skin. Accordingly, damage has to be indirectly assessed through a network of sensors able to feel the change in the structural behavior induced by the damage itself [11,12].

As the damage processes take place inside the structure, it would be physically-sound to also embed the SHM system into the composite structure [13,14,15,16]. This embedment can give rise to additional issues, as standard sensors are typically excessively large in size in comparison with the thickness of the (resin-enriched) regions between the laminae where they can be placed during the manufacturing stage. The local distortion of the microstructure they cause has been shown to result in a shorter lifetime, due to the inception of small defects that can eventually coalesce to provide a failure mode on their own. To avoid the SHM system being the source of a damage and so something that the system is supposed to feel and prevent as much as possible, in [17,18,19], we proposed to surface-mount inertial micro-sensors (MEMS) on composite plates; see also [20]. In fact, as composite structures are typically very thin and light in weight, the use of micro-sensors looks to be beneficial to attain the goal of minimal invasiveness of the SHM system.

The capability of detecting damage through an SHM system depends on at least the number, type and positions of the sensors. An appropriate choice of relevant design settings is fundamental to obtain an effective sensor network and to reduce its cost. As summarized in [21], well-established methods for the optimal sensor placement are based on the use of the Fisher information matrix or on energy principles; these methods are suitable for deterministic damage detection procedures. On the other hand, in order to take into account inherent uncertainties in the measurement process, system identification methods based on the Bayesian approach are instead needed; see, e.g., [12]. In this case, the optimal sensor configuration can be obtained by maximizing the difference in Shannon information between the probability distribution of damage estimation before and after the measurement process [22,23]. Despite the fact that these methods are suitable for model updating, they are characterized by a high computational cost; accordingly, we refer here to a deterministic approach only. The possible effects of measurement noise on the optimized sensor network configuration are then specifically investigated for a benchmark example, by showing the sensitivity to the said noise of the objective function ruling the deployment of the sensors.

By allowing for the difference in the length scales associated with the dimensions of the structure (macroscale), of the damaged area (mesoscale) and of the micro-sensors (microscale), we split the problem of optimal sensor placement into two stages, in a kind of top-down multiscale approach: the first, the top scale, deals with the identification of structural regions of higher sensitivity to damage; the second, the bottom scale, deals instead with the fine-tuning of the network deployment. The identifiability of damage location and amount is thus supposed to be enhanced by the mentioned tuning of the positions of all of the sensors in the network, collecting redundant measurements to compensate for measurement errors and noise.

The applicability of the offered approach to the SHM of lightweight composite structures is specifically investigated; the remainder of the paper is thus organized as follows. In Section 2 the multiscale (actually, two-scale) procedure for sensor deployment is detailed, starting from the concepts already introduced for a standard single-scale one; details relevant to the measurements collected with the sensors are provided, together with an explanation of how the topology optimizer can simultaneously handle all of the measurements and all of the possible damage locations. In Section 3, some results are provided: first, the exemplary case already tackled in [24,25], of a square plate characterized by a damage leading to a local reduction of the structural stiffness of unknown intensity and position, is considered; next, the more challenging geometry of a fuselage section with stiffeners is approached, to also show how the network topology can be affected by the type of damage (throughout the whole thickness of the composite shell, or localized inside as linked to delamination) to be detected. Results are provided not only in terms of optimal sensor deployment, but also in terms of the possible sensitivity of the deployment to the signal-to-noise ratio (varied by fictitiously tuning the external loads) and of the speedup in solving the optimization problem. Some concluding remarks are finally collected in Section 4, together with proposals for future related activities.

## 2. Optimal Sensor Deployment: A Multiscale Strategy

According to the procedure already introduced in [17,24,25], at each length scale, the proposed multiscale strategy rests on a formulation of topology optimization that deploys a pre-defined number of sensors over the structural element to be monitored. Accordingly, a scale-dependent objective function, defining the optimal spatial arrangement of the network, accounts for the damage-induced change in the structural response measured by all of the sensors.

Since damage location is unknown in principle and should indeed be identified [12], we assume that a structural degradation can be located anywhere. A direct measure of the aforementioned change in the structural response to the external actions can be obtained through a model of the structure, by performing a comparison between the response of the healthy configuration on one side and the responses of a set of supplementary configurations, each one featuring a stiffness reduction in a (small) region only, on the other side. The whole structure is thus subdivided into a number, say *n*, of non-overlapping regions (or sub-domains), each one small enough in comparison with the overall structural size so that the inner mechanical properties can be assumed to homogeneously and simultaneously vary in time. Thinking of a numerical (finite element based) approach to the problem of modeling the response to the external stimuli, *n* can be either considered as the total number of finite elements in the adopted space discretization (in the single-scale strategy) or as the number of sub-structures into which the component is divided (in the multiscale strategy); see, e.g., [26,27,28]. Each one of the mentioned supplementary structures is so considered undamaged, apart from a single element or domain where a scalar damage variable $0\leq d_{j}<1$, with j=1,...,n, is used to account for the degradation of the material stiffness: if *E* is the Young’s modulus of the virgin material, the stiffness in the damaged area reads $E_{j}=\left(1-d_{j}\right)E$. To properly allow for damage occurring anywhere, the same index *j* is used to denote one supplementary (fictitiously damaged) structure and the relevant damaged element/region in the mesh. Accordingly, the characteristic size of one element/region sets the resolution scale for damage assessment in a single-scale approach.

Moving to the mathematical formulation of the sensor deployment problem and following a rather conventional format of topology optimization (see, e.g., [29]), a field variable *x* is used as unknown to handle the placement of the sensors all over the design domain. Referring to the same finite element discretization introduced to compute the responses of the healthy and of the *n* supplementary damaged structures, such an unknown variable is discretized, as well, and smeared (or assumed constant) inside each element. The resulting discrete scalar field $0\leq x_{i}\leq 1$, with i=1,...,n, works as follows: if $x_{i}=1$, a sensor has to be placed over the *i*-th element to attain the optimal distribution of sensors constrained by the considered spatial discretization; if $x_{i}=0$, no sensors have to be placed over the *i*-th element; if $0<x_{i}<1$ for some values of *i*, it means that the constrained optimal solution does not allow one to place a pre-assigned number of sensors, say *N*, over the same number of elements. Since the solution is sought in the domain of real (not natural) numbers, it may then happen that the non-zero values of $x_{i}$, and so, the number of elements where sensors have to be placed, are greater than *N*, and some post-processing procedures are necessary to provide a sub-optimal, physically-sound sensor network topology. As discussed extensively in [17,24,25], a solution featuring $0<x_{i}<1$ for some values of the index *i* may be induced by symmetries in the structural geometry, and so, the aforementioned sub-optimal configuration can be straightforwardly devised. To avoid as much as possible the occurrence of no pure 0−1 solutions, i.e., of optima with intermediate values of $x_{i}$ (besides the mentioned cases linked to the problem geometry), a penalization is introduced in the formulation by replacing $x_{i}$ with $x_{i}^{p}$, where p>1 (for instance, p=2 as used in the simulations discussed next); see [24] for additional details on the relevant algorithmic formulation.

As originally proposed in [24], the objective function ruling sensor deployment can be built by handling all of the measurements by sensors placed where $x_{i}\neq 0$ and maximizing the sensitivity of a norm of such measurements either to the amplitude or to the location of damage. The first approach has a tendency to place the sensors where a larger change in the structural response can be felt, independently of damage location; the second approach provides instead a placement where the average signature of a damage located anywhere is higher. The second approach has been considered safer for SHM purposes, since the mentioned larger change in the response finally ruling the placement might not be activated in real cases, and so, the overall efficiency of the network can get spoiled; the other way around, maximizing the average change in the felt response instead of the maxima, allows one to implicitly (or approximately) take care of the signal/noise ratio of the sensors to be adopted in practice. Through the latter approach, the optimization problem for a single-scale approach reads:

(1) $$\left\{\begin{array}{c} \;\max_{x_{i}}\psi =\sum_{j=1}^{n}\sum_{i=1}^{n}\frac{x_{i}^{p}∥w_{ji}-w_{i}∥}{\max_{i}x_{i}^{p}∥w_{ji}-w_{i}∥}\\ s.t.\;\;0\leq x_{i}\leq 1\;\mathrm{and}\;\sum_{i=1}^{n}x_{i}\leq N\; \end{array}\right.$$

In Equation (1): ψ is the objective function to be maximized, which quantitatively defines the discussed overall change in the structural response felt by the whole sensor network; accordingly, *i* is the index running over the elements in the space discretization and adopted to locate the sensor, whereas *j* is the additional index denoting the damaged element in the *j*-th supplementary problem. For a fixed external loading condition, used for all of the n+1 structural analyses, $w_{i}$ stands for the response (in terms of, e.g., measurable displacements and rotations, or accelerations in the case of dynamics) of the healthy structure in the *i*-th element, and $w_{ji}$ is the response computed for the same *i*-th element in the *j*-th supplementary problem; the term $∥w_{ji}-w_{i}∥$ then stands for the norm of the variation of the structural response measured in the *i*-th element for a damage located in the *j*-th element. In the objective function ψ, each one of these variations is weighted by term $x_{i}^{p}$ and scaled by the term at the denominator, which is introduced so that $\frac{x_{i}^{p}∥w_{ji}-w_{i}∥}{\max_{i}x_{i}^{p}∥w_{ji}-w_{i}∥}\leq 1$ and so to handle the sensitivity to damage location as explained above. A limited amount *N* of sensors is allowed for, as prescribed by the constraint enforced on the sum of the unknown variables all over the design domain.

The above formulated constrained optimization problem can be efficiently solved through methods of sequential convex programming; see [24]. This kind of algorithm is well-suited to cope with large-scale problems [30], which can arise in the case of large values of *n*. However, the computational costs of the optimization procedure dramatically increase with *n*, keeping in mind that both the computing time of each single analysis providing $w_{i}$ or $w_{ji}$ and the number of supplementary problems to solve are set by *n*. Hence, for standard single-scale problems, a trade-off between solution accuracy (also ruled by the structural complexity) and computing time can define a reference value of *n*, or the characteristic size of the space discretization to be adopted. As already detailed in the Introduction, the design of a pervasive network of sensors (especially of micro-sensors) in structures of a complex geometry and of large dimensions provides the motivations to attack the problem of optimal deployment through a multiscale strategy.

**Figure 1:**
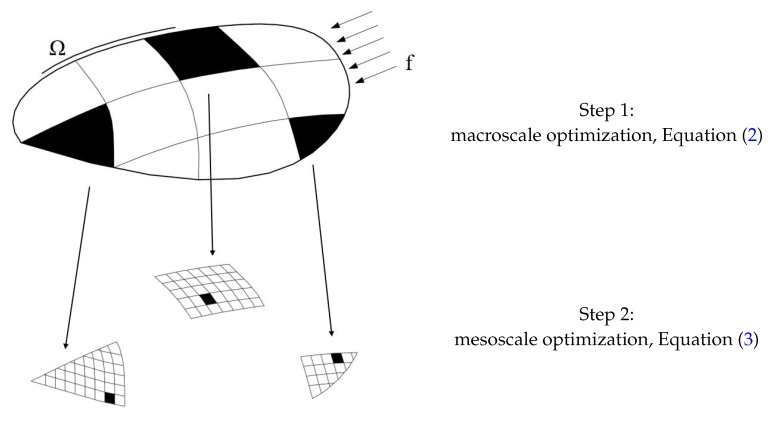
Graphical representation of the proposed optimization strategy.

To overcome the mentioned computational bottleneck, which is related only to the design stage of the sensor network according to the formerly-proposed single-scale deployment strategy, a multiscale approach can be conveniently implemented. Within a multiscale procedure, both *n* and *N* can be independently varied at each length scale: for instance, in a two-scale procedure, parameters $n_{M},N_{M}$ and $n_{m},N_{m}$ can be respectively introduced at the macroscale and at the mesoscale. In this way, the space discretization governed by $n_{M}$ and $n_{m}$ can be adapted to cope with, e.g., structural details irrelevant at the macroscale and instead of importance at the mesoscale if giving rise to a local stress intensification and so to a possible stress-governed damage inception and growth. On the other hand, as far as indices $N_{M}$ and $N_{m}$ are concerned, it can be seen that $N_{M}$ allows identifying a number of domains within which sensors have to be primarily deployed to assure a global high sensitivity of measurements to damage, and $N_{m}$ later allows one to fine-tune the positions of the available micro-sensors in the regions already identified at the macroscale. The whole procedure is schematically represented in Figure 1.

It must be noted that the reduction of the computational time attainable by such a multi-scale procedure is not used as a figure of merit while solving the optimization problem. As detailed in the formulation below, the actual goal of the procedure is the deployment of the sensor network handling accurate models of the structure at each length scale. Accordingly, the obtained speedup has to be considered as a positive side-effect of the multi-scale strategy.

The optimization problem in Equation (1) is thus first solved at the macroscale, over a discretization of the whole structure consisting of $n_{M}$ finite elements. At this scale, the problem reads:

(2) $$\left\{\begin{array}{c} \;\max_{x_{Mi}}\psi_{M}=\sum_{j=1}^{n_{M}}\sum_{i=1}^{n_{M}}\frac{x_{Mi}^{p}∥w_{ji}^{M}-w_{i}^{M}∥}{\max_{i}x_{Mi}^{p}∥w_{ji}^{M}-w_{i}^{M}∥}\\ s.t.\;\;0\leq x_{Mi}\leq 1\;\mathrm{and}\;\sum_{i=1}^{n_{M}}x_{Mi}\leq N_{M}\; \end{array}\right.$$

where $x_{Mi}$ are the optimization unknowns at the macroscale; $w_{i}^{M}$ and $w_{ji}^{M}$ respectively denote the macro-structural responses in the *i*-th element, for the healthy structure and for the $n_{M}$ damaged structures.

As discussed in [17,25], the results of Equation (2) turn out to be almost independent of the shape of the damaged area, provided that the finite element discretization is able to appropriately describe the deformation field in the region surrounding the damaged one. This means that the characteristic size of the $n_{M}$ elements should be smaller than or on the same order of the size of the region wherein damage may occur.

Next, each region for which the optimization problem (2) provides a non-zero $x_{Mi}$ value is discretized at the mesoscale with a mesh of $n_{m}$ elements. At this scale, to allow the aforementioned fine-tuning of network deployment, the characteristic size of the finite element is governed by the size of micro-sensors. The mesoscale problem is thus formulated as:

(3) $$\left\{\begin{array}{c} \;\max_{x_{mk}}\psi_{m}=\sum_{j=1}^{n_{M}}\sum_{k=1}^{n_{m}}\frac{x_{mk}^{p}∥w_{jk}^{m}-w_{k}^{m}∥}{\max_{k}x_{mk}^{p}∥w_{jk}^{m}-w_{k}^{m}∥}\\ s.t.\;\;0\leq x_{mk}\leq 1\;\mathrm{and}\;\sum_{k=1}^{n_{m}}x_{mk}\leq N_{m}\; \end{array}\right.$$

where $x_{mk}$, $k=1,...,n_{m}$, are the optimization unknowns at the mesoscale; $w_{k}^{m}$ stands for the structural response in the *k*-th element of the mesoscale mesh for the healthy structure; $w_{jk}^{m}$ refers to the structural response in the same *k*-th element for a structure damaged in the *j*-th element of the macroscale mesh. Due to scale-dependence, $w_{k}^{m}$ and $w_{jk}^{m}$ are computed by applying a suitable set of boundary conditions along the borders of the region analyzed at the mesoscale, to be used in conjunction with the external loads acting on the same region. Adopting a displacement-based finite element scheme both at the macroscale and at the mesoscale, this can be straightforwardly achieved by enforcing, along the outer boundary of the regions tackled by Equation (3), the displacement field there obtained as the solution of the problems at the macroscale.

As the interest is here in optimizing the deployment of the network and not in modeling damage evolution, at each length scale, elastic analyses are performed, with a region/element deficient in its stiffness for the auxiliary structures. Accordingly, a top-down uncoupled multiscale approach proves efficient to provide the kinematics of deformation along the borders of the meso-regions where sensors have to be placed.

To explicitly define what the entries in vectors $w_{i}^{M}$ and $w_{ji}^{M}$, $w_{k}^{m}$ and $w_{jk}^{m}$ have to represent of the structural responses, the sensors adopted should be taken into account; see e.g., [11,31]. As already pointed out in the Introduction, we refer here to lightweight structures like thin plates and shells, whose kinematics is mainly described by the displacement in the direction perpendicular to their (either flat or curved) mid-plane and by the rotations of segments perpendicular to such a mid-plane. For layered composite panels, the kinematics is usually more complex, due to the different mechanical response of typically anisotropic layers featuring their own spatial orientation; nevertheless, simplified solutions can still be obtained by managing the aforementioned kinematic variables only; see [32]. If sensors are supposed to be three-axis accelerometers, their capability to sense the gravity acceleration, and so their orientation relative to the gravity direction, can be exploited to sense the damage-induced change in the local rotation of the structural mid-plane. Accordingly, each vector representing the macroscale or mesoscale response to the external actions is assumed to collect the rotations about the axes of any local reference frame (typically one with two axes belonging to the mid-plane of the panel and one in the direction perpendicular to it), smeared at the element level.

Numerical simulations provided next will show the advantages of the proposed multiscale approach, in comparison with a single-scale optimization involving $n_{M}\cdot n_{m}$ discretization elements and problem unknowns.

## 3. Results

In this section, we present results related to the monitoring of damage for two rather different structures: the first one is a square plate fully clamped along its boundary and carrying a distributed load perpendicular to its mid-plane; the second one is a model of a stiffened composite aircraft fuselage, which can be affected by damage events either in the composite panel or in the stiffeners.

The first problem has been already approached with the single-scale strategy in our former investigations; see [12,17,24,25]. Referring to it once more has a three-fold purpose: to assess the effect of length scale interaction on the results, as related implementational aspects may introduce some artifacts in the results; to assess the reduction of the computational costs provided by the multiscale strategy, in comparison with the single-scale one; to show how measurement noise can be dealt with and affect the results of the methodology described in Section 2.

The second problem is instead of interest due to the multiple damage scenarios allowed for. The goal in this case is to show how the optimal network topology can be modified by the need to monitor damage events occurring throughout the whole thickness of the laminate or nested inside. Needless to say, the solution for real-life cases should provide the highest possible sensitivity to the damage, independent of location and type, under different loading conditions; accordingly, redundancy in the collected data helps to allow for all of the possible scenarios foreseen.

In both cases, it is supposed that three-axis MEMS accelerometers that have to be deployed feature an in-plane characteristic size ℓ=2.5 mm; see, e.g., [33]. Finite element simulations have been carried out with the commercial code Abaqus [34], using S8R eight-node shell elements with reduced integration.

### 3.1. Sensor Placement over a Thin Plate

As anticipated, we refer first to a case already considered in our former analyses due to its simple geometry and kinematics of deformation under any loading; see [12,17,25]. A thin, square plate was clamped along its whole boundary and subject to a distributed load all over its in-plane surface. The characteristic sizes linked to the three length scales of the problem were set as follows: the side length of the plate, representative of the macroscopic characteristic size, was L=1 m; the size of the damaged region was l=5 cm; and the size of the micro-sensors was, as said, ℓ=2.5 mm.

As reported in [17,25], a rather coarse mesh of 20×20 quadratic shell elements was used to discretize the plate at both scales, also in compliance with the ratios $\frac{L}{l}$ and $\frac{l}{\ell }$ between the dimensions reported here above. As far as the type of loading is concerned, it has been already shown that sensor deployment turns out to be almost independent of it, since a concentrated force in the middle of the plate (see also [12]) would induce a very similar sensor arrangement. Results turned out to be also independent of the shape of the damaged area [35], as long as the space discretization was able to appropriately capture the deformation field in the region surrounding the damage; accordingly, it can be assumed that any damage spread over a region larger than the mesoscale characteristic length can be appropriately handled.

By allowing for three-axis inertial MEMS accelerometers as micro-sensors to be placed, since they are designed to also sense the gravity acceleration, any rotation of the mid-plane of the plate induced by loading and damage (if any) can be measured through the sensed components of the gravity acceleration in a local reference frame attached to the sensor and so to the plate. To allow for the symmetry in the solution, which is mainly ruled by the problem geometry, the rotations about the two axes of a Cartesian reference frame belonging to the mid–plane of the plate were handled. An overall measure of rotation, say ϑ, can then be built as the norm of the rotation vector.

**Figure 2:**
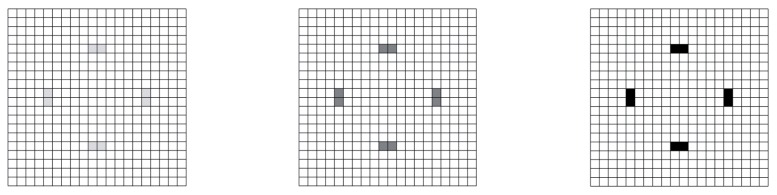
Plate problem, macroscale analysis: plane (top) view of the optimal sensor placement corresponding to (from left to right): $N_{M}=1$, $N_{M}=4$ and $N_{M}=8$.

For this problem, Figure 2 provides the optimal macroscale solution at a number of sensors to deploy varying in the range $N_{M}=1-8$. As shown by a different grey level at varying $N_{M}$, the optimizer always identified the same eight best locations that maximize the weighted sensitivities of the measured local values of ϑ to the damage. Such locations are in compliance with the mentioned symmetry in the problem solution; with $N_{M}=8$, the placement of a sensor over each single meso-area (or element) was attained.

**Figure 3:**
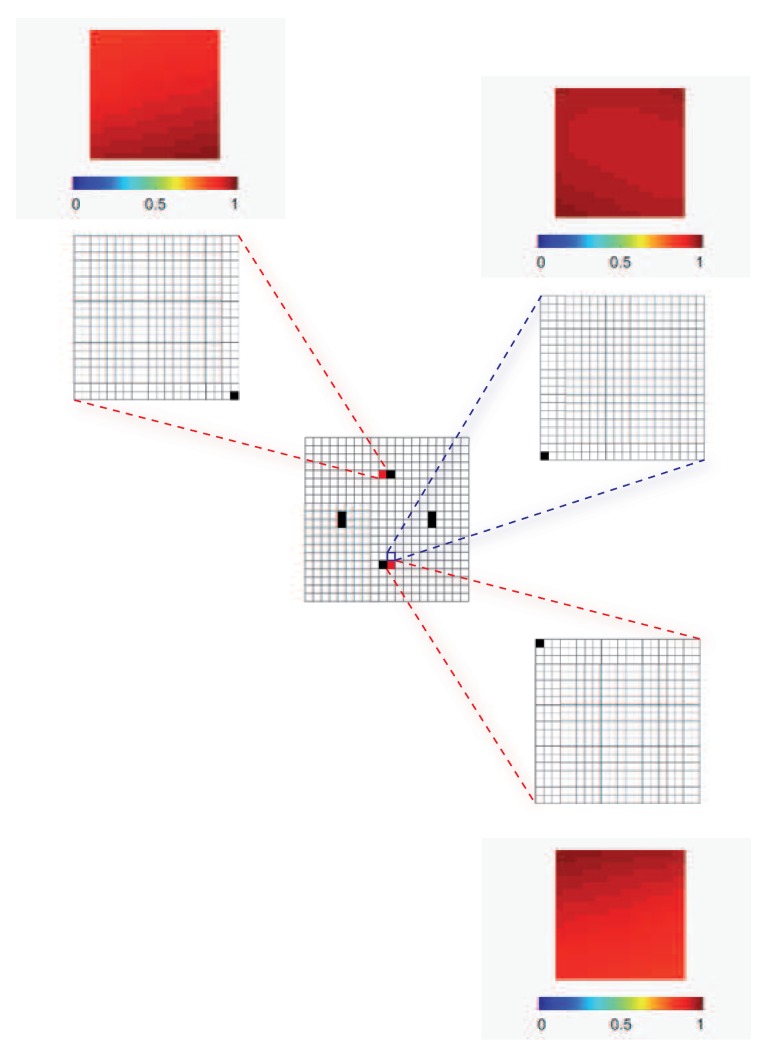
Plate problem, two-scale analysis: optimal sensor placement and mesoscale objective functions $\psi_{m}$, corresponding to $N_{M}=8$ and $N_{m}=1$.

Moving from the macroscale results reported in Figure 2, the mesoscale analysis has been next carried out by assigning $N_{m}=1$ for each region previously identified; hence, it has been assumed that only one micro-sensor has to be placed in every meso-region. In compliance with the problem symmetry at the macroscale, the analysis of one single meso-region would be sufficient to provide the final results in terms of micro-positioning. To further check the capability of the method to provide consistent outcomes, independently of the enforcement of symmetry in the solution, the placements corresponding to two opposite mesoscale regions are reported in Figure 3 (see the shaded red regions). In this picture, not only the best sensor micro-placement is depicted (as a black area), but also the sensitivity of ϑ to the damage, which amounts to one at most due to the already discussed scaling term in the objective function. As a further validation, the solution was sought in a purposely wrong region nearby the one provided by the macro-analysis (see the shaded blue area), to see if the optimizer moves the sensor to the location closest to the optimal one. As shown, it turns out that the optimal and the sub-optimal placements in the chosen domains were contiguous, so the procedure led to really effective outcomes. In Figure 3, the insets provide the micro-sensor placements over the mesoscale space discretization, whereas the localization of meso-regions featuring higher sensitivity to damage had been obtained using the macroscale space discretization. Due to the length scale ratios $\frac{L}{l}=\frac{l}{\ell }=20$, the space discretizations in the figure look all the same; indeed, even if a structured mesh of 20×20 shell elements had been adopted in all of the simulations, appropriate boundary and loading conditions had been always allowed for in each analysis, according to what was described in Section 2.

As for the speedup of the optimization procedure in comparison with a single-scale approach featuring the same resolution level *ℓ*, it must be noted that: with the two-scale approach 20×20+1=401, analyses were required at the macroscale, and 20×20+1=401 analyses were needed for each meso-region identified for placement (due to the symmetry, only one in the present case); with the single-scale approach 400×400+1= 160,001 analyses were instead required. Besides this aspect, by increasing the number of finite elements in the mesh and so the number of degrees-of-freedom (DOFs) of the problem, also the computational burden (CPU time) of each single analysis was increased, from about 0.2 s to about 49 s when run on a personal computer featuring an Intel Core i7-4790 CPU @3.60-GHz processor, 16 GB RAM and running Windows 10 64-bit as the operating system. Overall, the ideal speedup of the two-scale approach over the single-scale one can be computed as $\frac{160,001\times 49}{401\times 2\times 0.2}\approx$ 50,000; this value was somehow reduced by the post-processing of the results of the macroscale analyses, to set the appropriate input (in terms of boundary conditions) for the mesoscale ones. Such impressive speedup can be well exploited if re-analyses become necessary at the network design stage to assess the effects on the topology and, on top of all, on damage identifiability of different sensor types and/or features.

To assess the sensitivity of the results of the deployment strategy to measurement errors, the macroscale problem had been considered, and a fictitious zero-mean Gaussian noise had been added to the model responses $w_{i}^{M}$ and $w_{ji}^{M}$. Within a stochastic environment, the placement of a sensor is substituted by the probability to place a sensor over one element of the discretization mesh. Following a Monte Carlo approach, the measurement noise was sampled from the probability density function $N(0,\sigma^{2})$, σ being the relevant standard deviation, and the optimal network configuration was obtained for each sample; then, the scattered results related to all of the samples (whose number had been set so as to attain convergence in the deployment probability distribution) were merged to obtain the final outcome of the optimization procedure. Referring to the digital three-axis accelerometer [36] as the sensor to be adopted, the aforementioned standard deviation σ was computed for the values of the sensor frequency bandwidth (FBW) reported in the datasheet (FBW=50,200,400,800 Hz), according to:

(4) $$\sigma =0.15\sqrt{FBW}\frac{g}{\sqrt{\mathrm{Hz}}}$$

As we had assumed to measure rotations from the sensed acceleration components, σ was converted into the standard deviation for rotation measurements as follows:

(5) $$\sigma_{\vartheta }=\arccos\;\frac{1\;g-\sigma }{1\;g}$$

**Figure 4:**
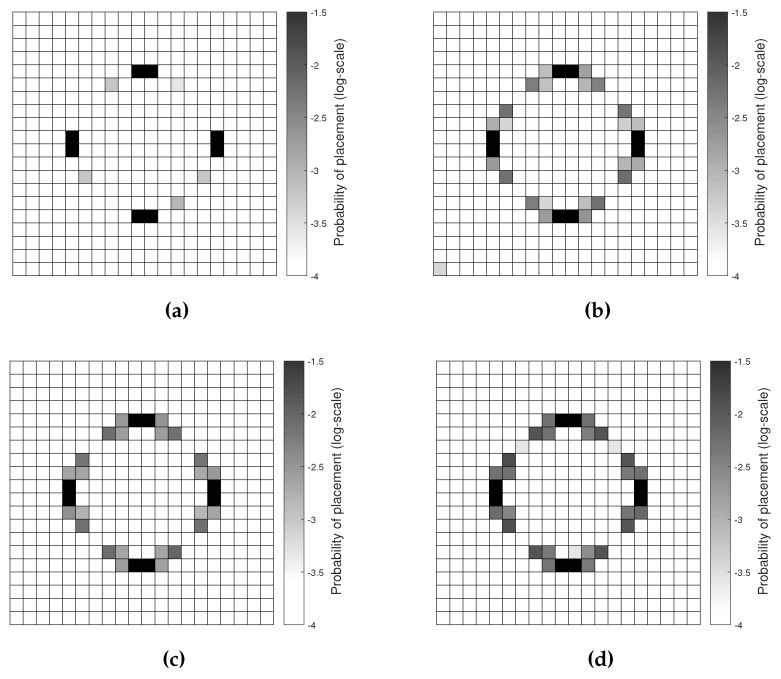
Plate problem, macroscale analysis, $N_{M}=8$: probability of (optimal) sensor placement considering (**a**) FBW=50 Hz; (**b**) FBW=200 Hz; (**c**) FBW=400 Hz; and (**d**) FBW=800 Hz.

In Figure 4, the resulting probability of sensor placement is shown at varying values of FBW and, therefore, of standard deviation σ. As expected, by increasing the measurement noise, the plot of the optimal configuration became more and more blurred, becoming different from the purely deterministic solution of Figure 2. Nevertheless, the most probable positions (namely, the highest values of placement probability) turn out to be independent of the measurement noise.

### 3.2. Sensor Placement over a Stiffened Fuselage Section

**Figure 5:**
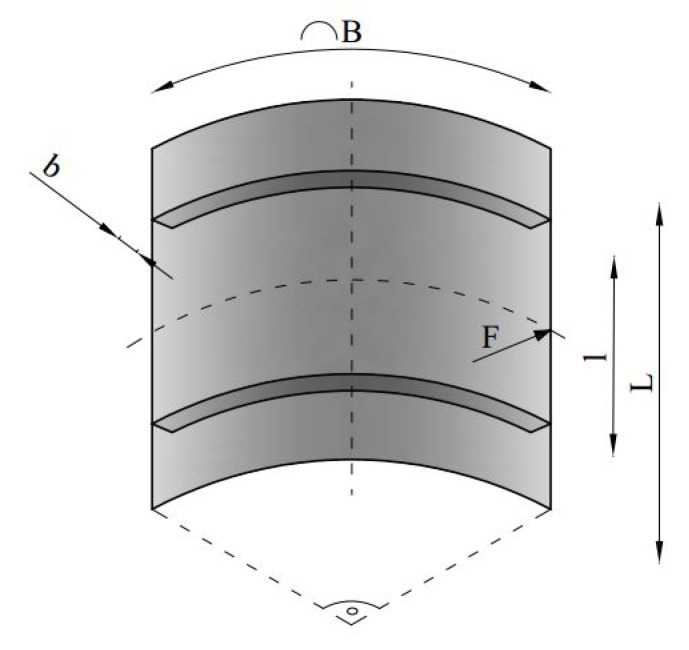
Stiffened fuselage section problem: geometry and notation.

To assess the capability of the proposed scheme in a more challenging situation, we refer now to the section of a stiffened aircraft fuselage described in [37]. The structural part consisted of an external composite shell and of two aluminum circular stiffeners; to slightly simplify the original geometry, we did not explicitly consider the window hole, which can be fictitiously allowed for as a part-through damage. Accounting for the loading condition envisaged in [37] and represented by a concentrated load *F*, only one quarter of the whole section can be considered in the analysis, and symmetry boundary conditions were applied along the vertical edges of the model in Figure 5. The relevant model dimensions are detailed in Table 1.

While in the plate problem, the structure had been supposed to be made of a homogeneous material and, so, the sensor deployment was not affected by the real elastic properties in the virgin state, in the present case, the solution can potentially depend on the mismatch in the stiffness of the composite and aluminum parts. The property values adopted in the simulations are reported in Table 2 and Table 3, for the composite material [38] and Aluminum 2024-O [39], where *E* is the Young’s modulus, ν the Poisson’s ratio and *G* the shear modulus. As far as the fiber-reinforced composite is concerned, the local reference frame linked to the values in Table 2 was assumed to have axis $x_{1}$ aligned with the fiber direction, axis $x_{2}$ transversal to it in the lamina plane (parallel to the mid-plane of the shell) and axis $x_{3}$ perpendicular to the shell mid-plane. Each ply of the laminate had been assumed to be 625 μm thick and the stacking sequence to be ${\left[90/0/\mp 45\right]}_{s}$. The fuselage was supposed to be free from geometrical imperfections, so perfectly cylindrical in shape.

**Figure 6:**
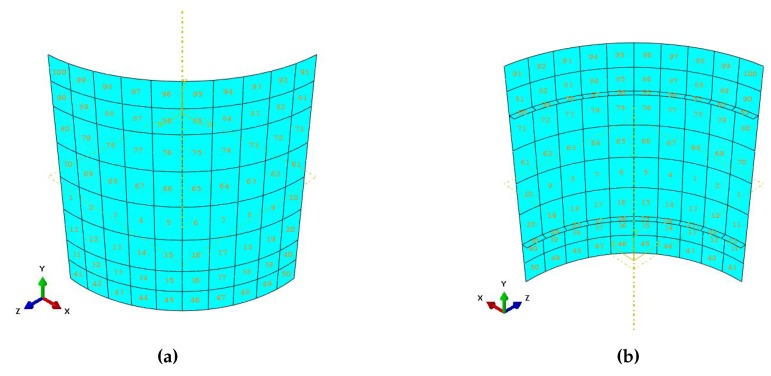
Stiffened fuselage section problem: (**a**) external and (**b**) internal views of the macroscale space discretization.

Figure 6 shows the internal and external views of the macroscale model of the analyzed structure, with the relevant element labeling (referring to the index *i* in Section 2), later used for the localization of the fine-tuning analyses. A rather coarse mesh consisting of $n_{M}=100$ elements only had been used. As before, a structured mesh had been adopted; although not shown for brevity, structured meshes had been adopted at the mesoscale as well.

As for the damage allowed for in the analysis, two different scenarios had been envisaged: (1) a part-through damage, with a reduction of the Young’s moduli $E_{11}$ and $E_{22}$ of all of the plies, to model the effects of impacts or strikes from the outer surface of the fuselage, or of aging; (2) a damage affecting Young’s modulus $E_{11}$ of Plies #4 and #5 only, to approximately simulate a delamination inside the laminate.

**Figure 7:**
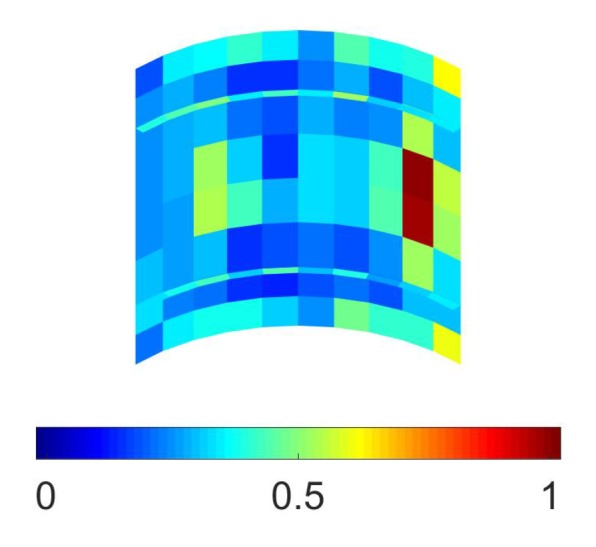
Stiffened fuselage section problem, Case (1), macroscale analysis: contour plot of the objective function $\psi_{M}$.

**Figure 8:**
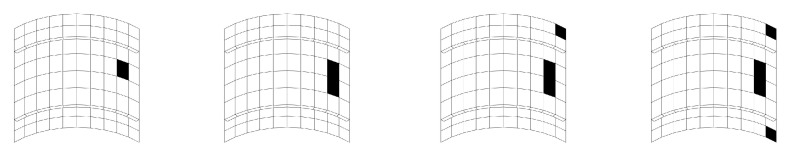
Stiffened fuselage section problem, Case (1), macroscale analysis: optimal sensor placement corresponding to (from left to right): $N_{M}=1$; $N_{M}=2$; $N_{M}=3$ and $N_{M}=4$.

Concerning Case (1), the contour plot of the relevant objective function $\psi_{M}$ is shown in Figure 7: as expected, the sensitivity turned out to be higher near the point of application of the load, where a local variation of the response induced by damage became enhanced. The results of the macroscale optimization procedure are collected in Figure 8 at a varying value of $N_{M}$: by enforcing the procedure to always place a sensor over a single element, so to have a pure 0–1 solution, it is shown that optimal placements were correctly obtained where the values of $\psi_{M}$ were higher, in compliance with the structural and loading symmetries for even $N_{M}$ values.

**Figure 9:**
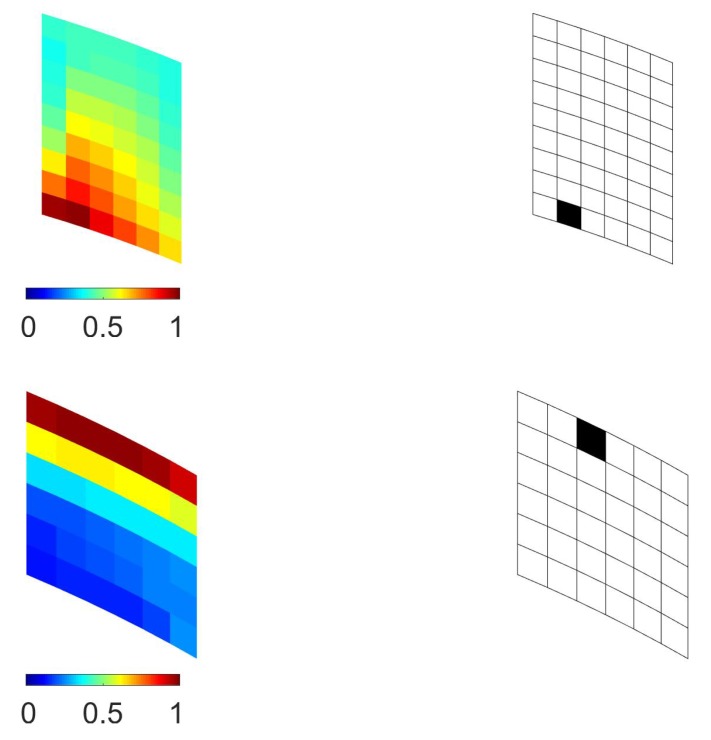
Stiffened fuselage section problem, Case (1), mesoscale analysis: (**left**) contour plots of the local objective function $\psi_{m}$, and (**right**) relevant optimal sensor placement with $N_{m}=1$; results reported for (**top**) i=69 and (**bottom**) i=100.

The outcomes of the subsequent optimization scheme at the mesoscale are shown in Figure 9, in terms of the contour plot of the objective function $\psi_{m}$ and of the relevant deployment of a single micro-sensor (namely, for $N_{m}=1$). These results were related to the two elements identified at the macroscale in the top half of the structural model; mirrored results would be obtained for the corresponding ones in the bottom half, due to symmetry.

**Figure 10:**
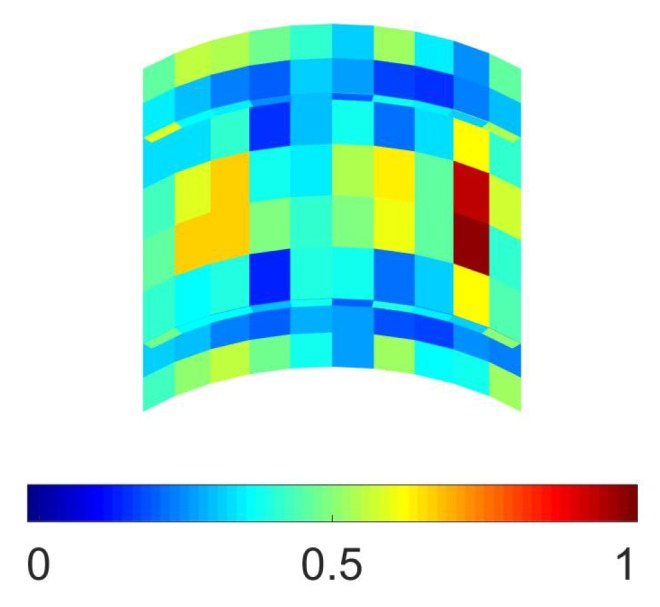
Stiffened fuselage section problem, Case (2), macroscale analysis: contour plot of the objective function $\psi_{M}$.

**Figure 11:**
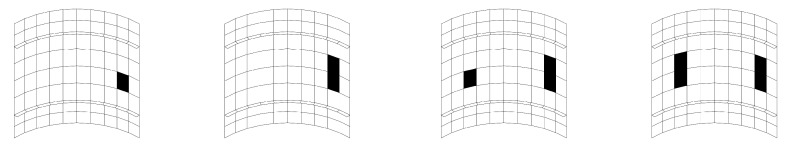
Stiffened fuselage section problem, Case (2), macroscale analysis: optimal sensor placement corresponding to (from left to right): $N_{M}=1$; $N_{M}=2$; $N_{M}=3$ and $N_{M}=4$.

**Figure 12:**
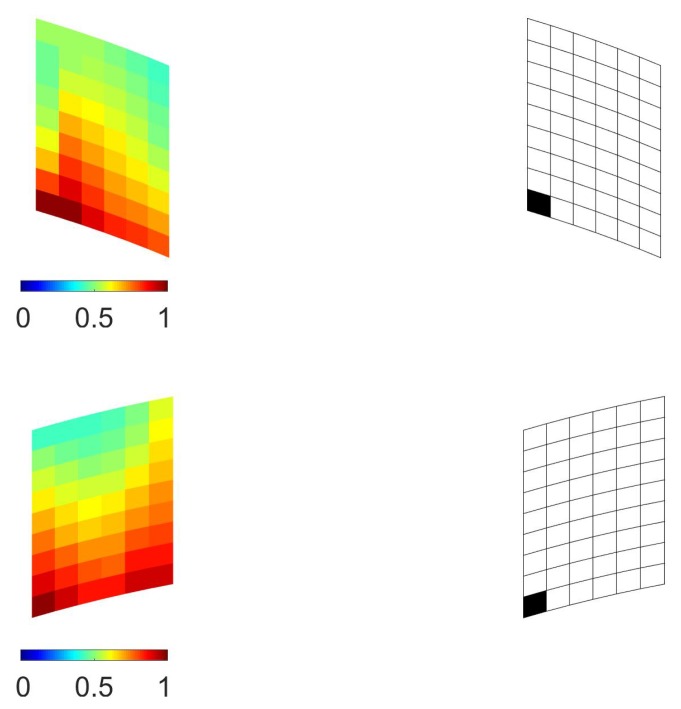
Stiffened fuselage section problem, Case (2), mesoscale analysis: (**left**) contour plots of the local objective function $\psi_{m}$, and (**right**) relevant optimal sensor placement with $N_{m}=1$; results reported for (**top**) i=69 and (**bottom**) i=63.

Concerning Case (2), the results of the multiscale investigation are collected in Figure 10, Figure 11 and Figure 12. As for Case (1), the objective function $\psi_{M}$ provided higher sensitivities close to the point of application of the external load, but at variance with the previous case, it is shown in Figure 11 that sensor placements were all located in the region between the two stiffeners, even for $N_{M}=4$. Subsequent mesoscale analyses provided the results of Figure 12, where $N_{m}=1$ had been adopted as before.

As anticipated, the network topology turned out to be somehow affected by the kind of damage considered for the layered external panel of the fuselage, even under the same loading condition. This feature, not reported for isotropic and homogeneous materials (see also [17,24]), thus required a further post-processing stage to cope with all of the envisioned damage events: alternatively, the solutions corresponding to Cases (1) and (2) can be merged to finally give the optimal topology to detect any kind of damage.

Referring finally to the computational complexity of the procedure, data are collected in Table 4 in terms of the total number of finite element analyses to run and the relevant number of DOFs per model. The comparison between the single-scale and the multiscale approaches is provided as in Section 3.1 at constant resolution length *ℓ* for micro-sensor placement. Concerning the CPU times required for the simulations, they respectively amounted to 3.3 s for each single-scale (fine mesh) analysis, 0.2 s for each macroscale analysis and 0.1 s for each mesoscale analysis. Overall, the resulting CPU times required to run all of the analyses for optimization purposes are gathered in Table 5, and so, a theoretical speedup exceeding 200 was attained. As already stated, such a value was decreased in practice by the post-processing of the macroscale outcomes, needed to define the input for the mesoscale simulations.

## 4. Conclusions

In this paper, we have proposed a deterministic multiscale extension of a previously-proposed topology optimization approach to deploy inertial micro-sensors over flexible, lightweight structures to be monitored. The optimization procedure was designed to maximize the sensitivity to damage of measurements collected through the sensor network, where damage has been considered as a local reduction of the stiffness of the hosting structure. With a focus on pervasive sensor networks, the objective function mathematically describing the aforementioned sensitivity has thus been assumed to be a weighted average of the damage-induced change in the structural response to the external stimuli, felt by all of the deployed sensors. Constraints additional to the maximum number of sensors to handle, like, e.g., the cost of the entire network, have not been addressed in this study.

To properly account for small structural details and for the real size of micro-sensors for SHM purposes, the topology optimization procedure has been split into two stages: a macroscopic one, to identify the regions whose responses are primarily affected by the presence of a damage of any amount and location; a mesoscopic one, to fine-tune the position of the micro-sensors within the regions identified at the macroscale. In this way, three length scales can be properly addressed in the analysis and optimization procedure, from the structural one (macroscale) down to the micro-sensor one (microscale).

Results have been first discussed making reference to a flat plate suffering damage in a meso-region of unknown position over the mid-plane of the structure. By also allowing for measurement noise, the method has been shown to provide an optimal deployment of the sensors somehow filtering out or reducing as much as possible errors in their location induced by the noise itself. Next, the problem of identifying the optimal locations to detect damage in a composite aircraft fuselage section has been approached. Once again, the method has been shown to easily provide optimal locations by maximizing the sensitivity of measurements and by also allowing for different kinds of damage.

The current approach will be further enhanced in future activities in order to properly account for all of the possible uncertainty terms in a stochastic, rather than deterministic, way. Since this aspect will require to somehow re-state the framework, it has not been addressed here as it is out of the scope of this work.

## Figures and Tables

**Table 1:** Fuselage dimensions; see Figure 5.

Parameter	Value
*L* (mm)	1000
*B* (mm)	1000
*l* (mm)	600
*b* (mm)	30

**Table 2:** Mechanical properties of the composite material.

Property	Value
$E_{11}$ (GPa)	151
$E_{22}$ (GPa)	8.44
$\nu_{12}$	0.018
$G_{12}$ (GPa)	4.20
$G_{23}$ (GPa)	2.71
$G_{13}$ (GPa)	4.20

**Table 3:** Mechanical properties of Aluminum 2024-O.

Property	Value
*E* (GPa)	73.1
ν	0.33

**Table 4:** Stiffened fuselage section problem: approach-dependent number of analyses and relevant computational burden at constant resolution length *ℓ*.

Numerical Model	Number of Analyses	Number of Model DOFs
single-scale model	3720 + 1	68,430
macroscale model	100 + 1	2046
mesoscale model	100 + 1	1158

**Table 5:** Stiffened fuselage section problem: computational times.

Approach	CPU Time (s)
single-scale	12,276
multiscale	60.6
